# Neuro-Optometric Rehabilitation to Prevent Post-concussion Visual Complications: A Case Report

**DOI:** 10.7759/cureus.110496

**Published:** 2026-06-08

**Authors:** Matthew R Smith, Collin J Reitz, Cynthia Martone, He Liu, Prasad S Dalvi

**Affiliations:** 1 Biology, Gannon University, Erie, USA; 2 Urgent Care and Occupational Health, Concentra Urgent Care, Erie, USA

**Keywords:** concussion, mild brain traumatic injury, neuro-ophthalmology, neuro-optometry, prism glasses, visual impairment

## Abstract

A concussion is a mild traumatic brain injury (mTBI) caused by an insult to the brain from an outside force. It is a concerning health issue affecting all ages, sexes, and races. Diagnosing mTBI involves physical tests, as mTBI can't be seen through imaging, such as CT or MRI scans. Management of an mTBI is typically rest and over-the-counter medications; however, mTBI can cause complications such as photosensitivity and ocular dysfunction. In this context, this article presents a case report of a 15-year-old hockey player who was hit in the face by an elbow at high speed. The patient was initially diagnosed with a minor laceration and cleared to play. The following day, the patient developed standard mTBI symptoms. Early evaluations downplayed the mTBI severity, which led to worsening symptoms, notably visual impairment. The patient was prescribed amitriptyline, an off-label prescription, for insomnia, only to worsen his condition. The delayed proper treatment and therapy prolonged his recovery. Ultimately, neuro-optometry, physical therapy, and occupational therapy helped improve his vision and balance. The neuro-optometrist properly diagnosed binocular vision dysfunction (BVD) or strabismus and prescribed prism eyeglasses for vision therapy. This case emphasizes the risks of misdiagnosing mTBI, off-label prescriptions, and delayed vision therapy. It highlights the importance of prompt and accurate care by utilizing a team approach involving neuro-optometrists or neuro-ophthalmologists if there are visual symptoms during the acute phase of concussion.

## Introduction

A concussion or mild traumatic brain injury (mTBI) is seen frequently in both youth and adults [1]. mTBI is an insidious injury that has become very prevalent, especially in sports [2]. Apart from sports injuries, other leading causes of mTBI in the United States include falls, motor vehicle crashes, and assault [1]. During competitive sports and recreational activities, about 3.8 million mTBIs occur in the USA annually; however, about 50% go undiagnosed and unreported [2]. Trauma to the head during a concussion causes transient brain dysfunction. If the trauma is of a mild nature, the prognosis is usually good, and most patients experience complete resolution of symptoms. During practice sessions and games, coaches and athletes must be aware of any trauma or injury to the head and when a concussion is suspected. The “Lystedt Law” requires the immediate removal from play of youth athletes with suspected sport-related concussion and clearance by a healthcare professional before returning to play [3]. If an athlete is suspected or diagnosed with an mTBI, they cannot return to play that day and should be observed and managed by a competent healthcare practitioner. Before athletes are cleared to begin the sport or athletic activity, they should be free of symptoms or back to their baseline following a five-day “Return to Play” protocol [4-7].

Post-concussion syndrome occurs in about 30%-80% of patients suffering from mTBI, and 20% may become persistent [8]. In the majority of athletes, post-concussion symptoms resolve within one to two weeks, and prolonged post-concussion symptoms (lasting for more than three months) may require additional tests and treatment by a multidisciplinary team [8]. Post-concussion symptoms can lead to long-term neurodegenerative changes that can frequently cause headaches, migraines, and slow processing and reaction times [9]. Headaches are the most common post-concussive symptom, along with brain fogginess with mild cognitive difficulty affecting memory and concentration [5,8,10]. Apart from headaches, visual manifestations may occur frequently due to the mTBI [11]. Extensive cortical, subcortical, cerebellar, and brainstem networks are involved in visual processing, which makes visual function particularly vulnerable to diffuse axonal injury and neurometabolic disturbances following mTBI, and visual impairments remain under-examined [12,13]. Common visual symptoms include blurry vision, eye fatigue, difficulty focusing, reading, or tracking, double vision, convergence insufficiency, accommodative insufficiency, impaired saccades and smooth pursuits, binocular vision dysfunction (BVD), photophobia, and visual motion sensitivity [14,15]. These visual disturbances can manifest as reduced academic or occupational performance. Some of these visual symptoms occur due to abnormalities in eye movements and focus regulated by the oculomotor system of the brain. The oculomotor system ensures visual stability by keeping images focused on the retina of the eye, for which it coordinates the extraocular muscles via the brainstem and cortex. Any damage to the oculomotor system or disturbance in its function is referred to as an oculomotor dysfunction [9]. At present, objective vision-based diagnostic tools for immediate assessment of oculomotor dysfunction are not available, which can cause delays in detecting oculomotor dysfunction and treatment of visual symptoms [16]. Emerging scientific evidence suggests that neuro-optometric rehabilitation and vision therapy may improve accommodation, vergence, and oculomotor deficits in selected patients with mTBI [17,18]. Despite increasing recognition of post-concussion visual dysfunction, variability remains in screening, referral, and implementation of neuro-optometric rehabilitation, and detailed case reports describing individualized rehabilitation strategies remain limited. This case study presents a report on post-concussion oculomotor dysfunction and delayed assessment of visual function within 12 weeks of an mTBI injury that may be responsible for persistent neurological symptoms.

## Case presentation

A 15-year-old Caucasian male presented to the urgent care after getting struck just above the bridge of the nose with the elbow of another person at high speed while playing ice hockey, with a helmet. The patient suffered a small V-shaped laceration over the bridge of the nose. At the time of the injury, there was no evidence of ocular or periorbital trauma or any history of a brief loss of consciousness with retrograde amnesia, headache, disorientation, irritability, or unsteady gait. At the urgent care, the patient was cooperative, completed a neurologic examination, and reported no prior history of concussions. The laceration on the nose was cleaned and dressed, and the patient was given a clean bill of health and discharged. On the same day, hours later, the patient was allowed by the coach to play another hockey game. The next day, the patient was lightheaded, developed headaches, his legs felt weak, and he had difficulty with balance. Later in the week, the patient was told to see a sports-medicine physician for a possible concussion.

One week after the initial injury, the patient continued to suffer from the above-described symptoms and visited a sports medicine physician, where he was evaluated for a concussion. The results of numerous tests are presented in Table 1.

**Table 1:** Results of concussion tests.

	Category	Test	Findings
1	Coordination	Finger to nose (eyes open)	Normal
		Finger to nose (eyes closed)	Normal
		Rapid alternating hand motion	Abnormal
2	Oculomotor	Horizontal saccades	Abnormal
		Vertical saccades	Abnormal
		Convergence	Abnormal
		Extraocular muscle	Intact
3	Memory	Delayed recall performance	4/5
4	Balance	Rhomberg	Abnormal
		Heel-to-toe gait	Abnormal
5	Neurologic/cognitive assessment	Orientation	5/5

The patient was told he could return to school, and nothing stood out as being different than a run-of-the-mill two-week concussion. Playing ice hockey was the only thing the patient was advised to avoid, along with limiting screen time. 

Two weeks later, the patient continued to have symptoms with minimal change but started to improve slightly. Headaches were intermittent and not as severe as before. However, the patient started having trouble concentrating in the classroom, experiencing light sensitivity, sleeping more, and having difficulty reading and focusing. He attended school full-time, and the lights, noise, etc., in his class started bothering him constantly.

During the third week post-trauma, the patient started having increased difficulty with school activities as well as falling asleep at night. This prompted the physician to place the patient on 25 mg of amitriptyline to help with his symptoms and sleep better. The patient was referred to neuro-optometry for his visual symptoms, with a one-month wait for an appointment.

**Figure 1:**
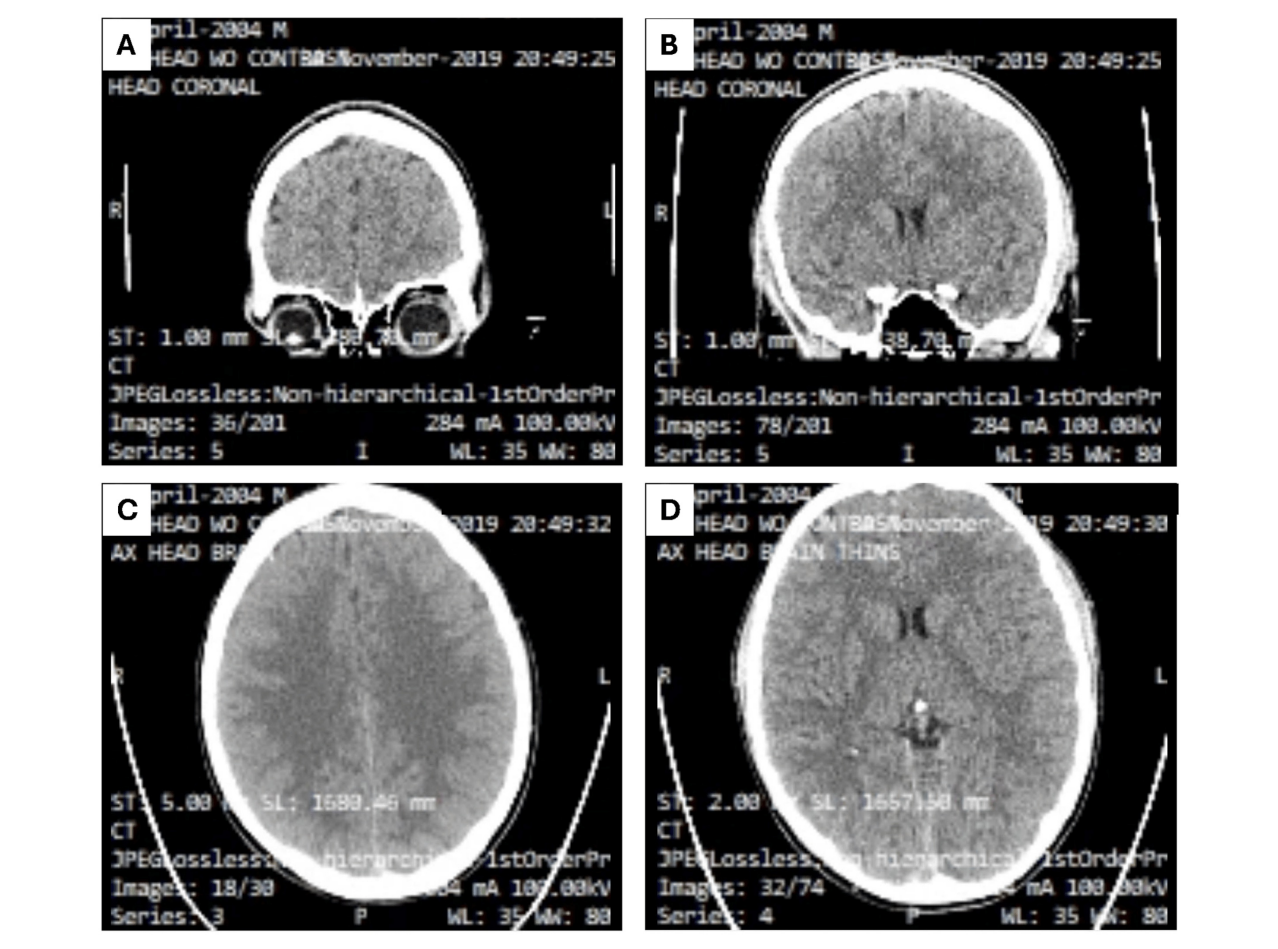
Computed tomography (CT or CAT) scan images. (A-D) Representative computed tomography (CT or CAT) scan images. No acute intracranial pathology was identified as there was no evidence of acute intracranial hemorrhage, midline shift, mass effect or extra-axial fluid collection. The skull base and calvarium were intact. The official radiology interpretation found no clinically significant abnormalities related to the patient's presentation.

The next week, when the patient returned to school while still on amitriptyline treatment, he began to act very strangely; he had no control of his body, and he was constantly swaying, almost falling out of his chair. Immediately after this episode of uncontrolled body movements, amitriptyline was discontinued, as it was suspected to be the cause of this episode. This episode also caused the patient to stop attending school for the time being and work on his classes from home with a home tutor. The tutor would visit the patient twice a week to help him with his work since he was having a great deal of pain while reading. However, despite discontinuing amitriptyline, the patient experienced three separate episodes of extreme personality changes lasting anywhere between two and four hours that went away on their own. The third episode, which was the longest, prompted a visit to the emergency room (ER). The ER doctor concluded this episode could have possibly been some sort of atypical seizure. A CT scan was performed, but nothing conclusive came back, even though the patient continued to have a wide array of symptoms (Figure 1). Regarding the possible asymmetry noted on the CT image in Figure 1, the official radiology interpretation found no clinically significant abnormalities related to the patient's presentation. An EEG and neurology consult were scheduled. 

**Figure 2:**
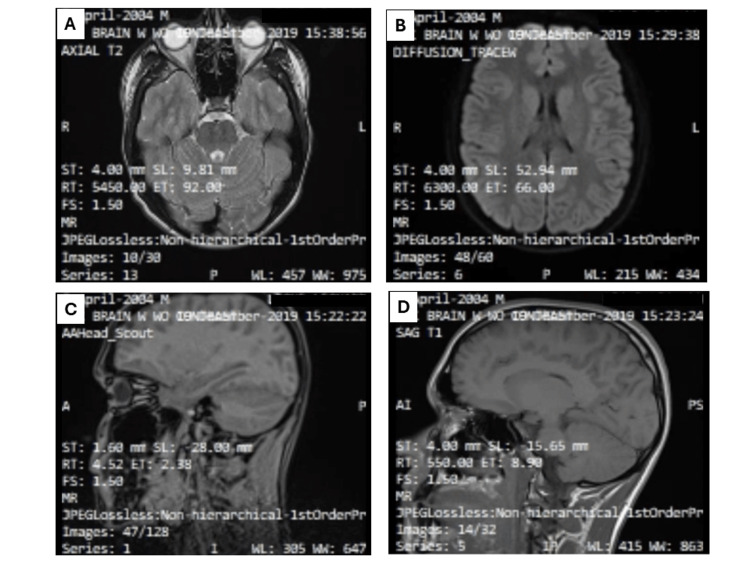
Magnetic resonance imaging (MRI) scan images. (A-D) Representative magnetic resonance imaging (MRI) scan images. There was no evidence of intracranial hemorrhage, mass effect, acute infarct, or abnormal intracranial enhancement. The official radiology interpretation found no clinically significant abnormalities related to the patient's presentation.

Into the fifth and sixth week post-mTBI, the patient’s symptoms did not improve and, if anything, worsened. The EEG was reported to be normal, prompting the neurologist to order an MRI that showed no evidence of intracranial hemorrhage, mass effect, acute infarct, or abnormal intracranial enhancement (Figure 2). 

Two and a half months later, the patient was still experiencing headaches, light sensitivity, sound sensitivity, nausea, fogginess, drowsiness, and trouble reading. Headaches occurred every one-two hours and were about a 4/10 on the pain scale. He developed a “very bad headache” once every two-three days. For his light sensitivity, he used all electronics on low brightness to reduce the strain on his eyes. His balance was still off, and he was having trouble walking in a straight line. He also had slight nausea at times, especially when trying to balance. Reading was still tough for the patient. The home tutor continued to come to his house two to three times a week.

After a long wait of about three months post-mTBI, the patient was seen by a neuro-optometrist. The neuro-optometrist noted the patient’s shoulders were uneven, causing him to walk at an angle. He also noted the patient’s BVD or strabismus. BVD occurs when the eyes are misaligned, forcing the eye muscles and brain to struggle constantly to fuse two different images into one clear picture. This was the most likely cause of his eye strain while reading and focusing. To counteract the BVD, the patient was given glasses with a two-prism base-up yoke and a 10% omega blue tint for the photophobia. The prism glasses worked to reset each shoulder’s height, to put his walk back in a straight line, and provide visual compensation for the midline posterior shift. The patient wore these prism glasses for the next two months. During this entire period, he attended a mixture of occupational therapy and physical therapy. Physical therapy focused on balance and conditioning, while occupational therapy focused on helping the brain work with the eyes. During occupational therapy, the patient did oculomotor training exercises such as focusing on an object coming closer to the eyes and staying focused on it as it moved farther away. There were a few techniques used to counteract the effects of a compressed visual field. One was “Dynavision” therapy, which is a large electronic board with light-up buttons that patients have to press to test visual and cognitive functions. Another was the neurosensory integrator (NSI), a large interactive touch screen that tests visual, cognitive, and motor functions by displaying moving objects, numbers, symbols, and lights, with the patient interacting with the screen using their hand or eye movements. As the patient continued to do the exercises, his condition started to slowly improve.

After going to each therapy twice a week for over two months, the patient finally began to feel better. He no longer had to wear the prism glasses or attend therapy. With most of the symptoms gone after nearly six months, the patient was finally able to return to normal activities. Although the patient was able to resume normal activities, five years after the trauma, he still suffers from minor light sensitivity and headaches when reading for too long. The patient continues to suffer from hypersomnia since his initial injury; however, his sleep has always been fragmented and never restful.

## Discussion

This case highlights the challenging process of diagnosing a concussion or mTBI and how the multifaceted approach should be considered in the treatment of a concussion or any mTBI. An mTBI is caused by a blow to the head, whether direct or indirect. Thus, the mechanism of injury is an important and critical component for diagnosis and treatment. If there is a history of past mTBI, the information is a key factor in predicting outcomes. Athletes who experienced a previous concussion are more likely to sustain a subsequent concussion and mTBI than those without a history of prior concussions [19]. To protect youth athletes from the consequences of mTBI, all 50 states in the USA currently have laws in place mandating concussion education for athletes as well as coaches. The “Lystedt Law” requires the immediate removal from play of youth athletes with suspected sport-related concussion [3]. Again, self-reporting of symptoms relies solely on the athlete [3]. Failure to recognize the signs and symptoms of an mTBI can lead to worsening symptoms, a repeat injury, and long-term consequences. mTBI signs, symptoms, and immediate actions should be known by all players, coaches, referees, staff, and parents. Red flag signs and symptoms include loss of consciousness for greater than a minute, vomiting, neurological abnormalities, seizures, headaches, skull fracture, and signs including raccoon eyes or Battle's signs, which are easy to identify. The athlete should be taken directly to an ER, not urgent care [20]. Symptoms such as a headache followed by dizziness are less likely to be recognized by an outside observer, which can lead to a prolonged recovery that is sixfold greater [3]. Additional subtle symptoms that are experienced internally by the athlete include balance problems, visual problems, disorientation, brain fogging, blurry vision, sensitivity to light and noise, and fatigue. All of which can be masked by the athlete to prevent being pulled from the game [20]. Responding quickly and correctly to a sports-related concussion or mTBI is of utmost importance to prevent further injury or long-term consequences and even death. Immediate removal from play after a possible mTBI incident is necessary because continued physical activity can result in a longer recovery period, increased risk for repeated concussions, and an overall poor outcome [3]. A coach’s knowledge about concussions, mTBIs, and their signs and symptoms can affect the attitudes and beliefs of the athletes on reporting mTBI symptoms [21]. The underreporting of symptoms by an athlete, family member, or coach in an attempt to continue to play presents challenges in diagnosing and creating a medical plan of action. Full disclosure of the incident and symptoms is imperative for the provider to develop a comprehensive treatment plan.

Common barriers that prevent athletes from reporting symptoms are a lack of education, which leads to the inability to identify their symptoms as being a possible concussion and realize the severity of the injury [3]. Also important is the invincible drive not to be removed from the game and to let the team down. However, having the knowledge and information on mTBI does not directly translate into reporting symptoms of a concussion. Behavior analysis research shows that behavior is more affected by short-term consequences rather than long-term consequences, and the knowledge about mTBI doesn’t always translate to reporting symptoms and seeking its immediate and proper management [21]. In this case report, the lack of proper education on concussion evaluation and management is clear, as the patient was sent back to resume playing ice hockey rather than being sent to an ER. This return to the game was apparently in contrast to the “Lystedt Law” that requires the immediate removal from play of an athlete with a suspected concussion [3]. Regarding the diagnostic investigations for mTBI, the decision to obtain a CT is based on the clinical assessment, judgment, and experience of the provider when diagnosing and ruling out intracranial injuries that may require urgent interventions from neurosurgery or a neurological evaluation. An initial CT scan has a 99.7 percent predictive value for excluding an injury requiring neurosurgical intervention [22]. An MRI is superior in detecting small brain contusions, small extra-axial hematomas, microhemorrhages, and small white-matter lesions that represent acute traumatic axonal and/or microvascular injury. Numerous studies have shown that a negative initial head CT is not a rule-out of an mTBI. Intracranial injuries have been discovered on MRI scans and used to predict disabilities in mTBI [23]. EEG is not indicated in most patients with post-concussion syndrome [24]. There are no unique features of an mTBI that either an EEG or quantitative EEG would identify [24]. In the present case report, the patient’s CT scan, EEG, and MRI were all negative. Despite no evident brain trauma, the patient continued to have a wide array of symptoms, such as headaches, light sensitivity, sound sensitivity, nausea, fogginess, drowsiness, and trouble reading for around two and a half months after suffering the trauma.

Earlier, during the third week post-trauma, the patient in this case report was treated with 25 mg of amitriptyline for symptoms and sleep disturbances. During the treatment period, the patient began to act very strangely, having no control of his body as he was constantly swaying, almost falling out of his chair. These adverse events prompted the physician to discontinue amitriptyline. Amitriptyline is a tricyclic antidepressant that is used for off-label purposes 81% of the time, including headache in mTBI; however, the research is inconclusive on the use of amitriptyline to address headache in mTBI [25]. A number of studies reveal beneficial effects; however, the patient presented in this case did not tolerate it well. As such, studies have shown an increased risk of suicidal thoughts and behavior in children, adolescents, and young adults. Patients started on antidepressants should be monitored and observed for worsening of clinical signs [26].

An mTBI is usually diagnosed and treated in the emergency department or urgent care, and at rare times by family practice providers. Once a diagnosis has been made, follow-up care is maintained or prolonged through family practice providers, neurologists, and chiropractors, and often leads to tertiary referrals with neurorehabilitative specialists [27]. It is known that mTBI may cause disturbances in the brain and may lead to a variety of symptoms, such as vestibular and visual impairments, with common complaints of blurred or double vision, eye fatigue, photophobia, and the inability to focus. Ocular alignment and coordination of binocular movement may be a key feature of a concussion. However, strong evidence is lacking in the research and test development for visual impairments that can be used as a reliable diagnostic tool for concussion or mTBI. Ocular motor- and vision-related therapies immediately following an mTBI are anecdotal in evidence and do not include emerging evidence to support their effectiveness [28]. However, it is evident that the patient in this case report benefited from the neuro-optometrist therapy three months post-injury. The neuro-optometrist prescribed prism glasses that counteracted both eyes working against each other. He wore the prism glasses for the next two months and simultaneously received occupational and physical therapy for vision and balance correction. As the patient continued to receive these therapies, his condition started to slowly improve. In this context, some published data suggest that vision therapy can be an effective intervention for mTBI [29]. Regarding this case report, we acknowledge that, given that this is a single uncontrolled case report, there is a possibility of spontaneous post-concussion recovery over time, particularly since the patient improved gradually over several months while receiving multidisciplinary rehabilitation.

Though there are some helpful treatment guidelines for post-mTBI, there are no evidence-based clinical guidelines or best practices that are specific to the United States [30]. Due to the multiplicity of the presentation of mTBI symptoms, no one-treatment approach will meet the needs of every patient. Therefore, many clinicians have accepted the benefit of a team-based approach, even though not all empirical research supports this model. Encouragingly, data have shown quicker recovery times, and reduced symptoms have occurred when a team of healthcare providers is involved in the diagnosis and treatment during the acute phase of concussion or mTBI [31]. In the case presented here, an early intervention by a neuro-optometrist or neuro-ophthalmologist and occupational and physical therapists during the acute phase of the mTBI may have shortened the recovery times and prevented lingering long-term effects. Thus, more research, clinical trials, and education on utilizing a team approach involving a neuro-optometrist or neuro-ophthalmologist, if there are visual symptoms during the early phase of an mTBI, are warranted.

## Conclusions

Apparently, the visual system seems to be an often-neglected component of a concussion or mTBI treatment plan; however, if there are visual symptoms during the acute phase of an mTBI, visual testing should be added to the mTBI treatment plan. Through the use of neuro-optometric rehabilitation, the vision system can be retrained, and visual symptoms that may stem from a TBI can be resolved or even eliminated. This can occur through the use of eye-training exercises to rewire the brain and enhance visual function. As of now, there is not a single diagnostic test that is comprehensive for all visual deficits post-mTBI. Patient symptoms should guide specific testing of the visual system. In the present case, if the patient had received proper neuro-optometric, physical, and occupational therapies during the acute phase post-mTBI, it may not have taken six months to be nearly symptom-free post-traumatic head injury. However, we acknowledge that since the patient improved gradually over several months while receiving multidisciplinary rehabilitation, there is a possibility of spontaneous post-concussion recovery over time. More clinical trials are needed to emphasize early visual examination and treatment by a neuro-optometrist during the acute phase of the mTBI to prevent long-term visual impairments.
